# Ipsilateral Fixation and Reconstruction of the Cervical Spine after Resection of a Dumbbell Tumor Via a Unilateral Posterior Approach: A Case Report and Biomechanical Study

**DOI:** 10.1111/os.13798

**Published:** 2023-07-11

**Authors:** Yongqiang Zeng, Zhiping Huang, Zucheng Huang, Yongquan Cheng, Qing'an Zhu, Wei Ji, Hui Jiang

**Affiliations:** ^1^ Division of Spine Surgery, Department of Orthopaedics, Nanfang Hospital Southern Medical University Guangzhou People's Republic of China

**Keywords:** Biomechanics, Bone Fusion, Dumbbell Tumor, Internal Fixation, Subcervical Spine

## Abstract

**Objective:**

There is lack of an internal fixation following resection of a dumbbell tumor by hemi‐laminectomy and facetectomy that achieves adequate stability with less trauma. Unilateral fixation and reconstruction (unilateral pedicle screw and contralateral lamina screw fixation combined with lateral mass reconstruction, UPS + CLS + LM) may be an ideal technique to address this problem. A biomechanical comparison and a case report were designed to evaluate its spinal stability and clinical effect.

**Methods:**

Seven fresh‐frozen human subcervical specimens were used for the biomechanical testing. The conditions tested were: (1) intact; (2) injured (single‐level hemi‐laminectomy and facetectomy); (3) unilateral pedicle screw (UPS) fixation; (4) UPS fixation combined with lateral mass (LM) reconstruction (UPS + LM); (5) UPS fixation and contralateral lamina screw fixation (UPS + CLS); (6) UPS + CLS + LM; (7) UPS fixation and contralateral transarticular screw fixation (UPS + CTAS); (8) bilateral pedicle screw (BPS) fixation. Range of motion (ROM) and neutral zone (NZ) were obtained at C5‐C7 segment under eight conditions. In addition, we report the case of a patient with a C7‐T1 dumbbell tumor that was treated by UPS + CLS + LM technique.

**Results:**

Except left/right lateral bending and right axial rotation (all, p < 0.05), ROM of UPS + CLS + LM condition in other directions was similar to that of BPS condition (all, p > 0.05). There was no significant difference between UPS + CLS + LM and the UPS + CTAS condition in other directions of ROM (all, p > 0.05), except in left/right axial rotation (both, p < 0.05). Compared to UPS + CLS condition, left/right lateral bending ROM of UPS + CLS + LM condition were significantly reduced (both, p < 0.05). UPS + CLS + LM condition significantly reduced ROM in all directions compared to UPS and UPS + LM condition (all, p < 0.05). Similarly, except lateral bending (p < 0.05), there was no difference in NZ in other directions between UPS + CLS + LM and BPS condition (both, p > 0.05). There was no significant difference between UPS + CLS + LM and UPS + CTAS condition in NZ in all directions (all, p > 0.05). Axial rotation NZ of UPS + CLS + LM condition was significantly reduced compared to UPS + CLS condition (p < 0.05). Compared to UPS and UPS + LM condition, NZ of UPS + CLS + LM condition was significantly reduced in all directions (all, p < 0.05). The patient's imaging examination at 3 months postoperatively indicated that the internal fixation did not move and the graft bone was seen with fusion.

**Conclusion:**

After resection of a dumbbell tumor in the cervical spine, UPS + CLS + LM technique is a reliable internal fixation method to provide sufficient immediate stability and promote postoperative bone fusion.

## Graphical abstract

After resection of a dumbbell tumor in the cervical spine, unilateral pedicle screw and contralateral lamina screw fixation combined with lateral mass reconstruction (UPS + CLS + LM fixation) is a reliable internal fixation method to provide sufficient immediate stability in the biomechanical study and promote postoperative bone fusion in the case study.

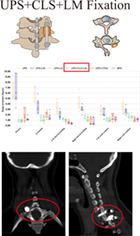

## Introduction

A dumbbell tumor is a lesion that connects two or more separate regions of the spine, such as the intradural, epidural, intervertebral foraminal, and paravertebral regions, and accounts for approximately 18% of all spinal tumors.^1^, ^2^, ^3^ Schwannomas are the most common pathological type of dumbbell tumor, with Eden type 3 tumors that involved the epidural and paravertebral regions being most common. About 44% of dumbbell tumors occur in the cervical spine and are mainly Eden type 2 tumors involving the intradural, epidural, and paravertebral regions.^3^ Removal of an Eden type 2 or type 3 cervical dumbbell tumor requires performing a hemi‐laminectomy and facetectomy using the posterior approach.^3^ However, damage to the lamina, muscles, ligaments, and facets increases the risk of subcervical instability. Therefore, internal fixation and fusion should be performed after posterior resection of a unilateral dumbbell tumor.^4^, ^5^

There are three methods for short segment spinal fixation, pedicle screw fixation, lateral mass screw fixation, and transarticular screw fixation, and each has its own limitations. Bilateral pedicle screw fixation is the gold standard for spine surgery instrumentation. However, with increased use and longer follow‐up time disadvantages of bilateral pedicle screw (BPS) fixation such as excessive soft tissue dissection, high risk of injury to neurovascular structures, stress shielding caused by strong fixation, and the potential for accelerating adjacent segment degeneration are being recognized.^6^, ^7^ On the other hand, unilateral pedicle screw (UPS) fixation may result in a higher incidence of instrumentation failure and pseudarthrosis.^8^, ^9^ Biomechanical studies have shown that both transarticular screw (TAS) fixation and lateral mass screw fixation have the same capability for stabilizing an Intact cervical spine.^10^ However, transarticular screw fixation constrains flexion‐extension less when compared to lateral mass screw fixation for the treatment of an unstable subcervical spine.^11^ In short, the clinical outcomes of these approaches still remain controversial due to the higher rates of adverse effects, including increased risk of neurovascular, increased dissection of soft tissues, or inadequate fixation of implants. However, these problems can be solved by using laminar screw fixation techniques in the subcervical spine. This method is associated with minimal surgical trauma, a reduced rate of complications, and providing reliable fixation strength compared with conventional fixation methods.^12^, ^13^

Bone graft fusion is an important method for internal fixation of the spine. Lateral mass (LM) reconstruction by graft bone can adequately transfer load‐bearing forces from adjacent segments to reduces the stress on screw and rod systems.^14^

During removal of a dumbbell tumor via a unilateral posterior approach, the damage to the posterior stabilizing structures, which include the lamina, facet joints, and posterior ligaments,^15^ has been shown to increase spinal instability, accelerate disc degeneration and progression to adjacent segment disease (ASD). In addition, surgical damage to cervical muscle attachment points can weaken the cervical stability.^16^ Therefore, internal fixation treatment should be considered to provide additional support for the stability of the affected segment. However, it is unclear if unilateral pedicle screw and contralateral lamina screw fixation combined with lateral mass reconstruction (UPS + CLS + LM) provides sufficient stability as compared to pedicle screw fixation and transarticular screw fixation after removal of a dumbbell tumor via a unilateral posterior approach.

This study was designed: (i) to compare the biomechanical stability of different instrumentation techniques after single‐level hemi‐laminectomy and facetectomy of the cervical spine; (ii) to report the results of a patient with a cervical dumbbell tumor who was treated with the UPS + CLS + LM technique.

## Materials and Methods

### Biomechanical Study

The study was approved by Medical Ethics Committee of Nanfang Hospital of Southern Medical University. The specimens were obtained from the Department of Anatomy, Southern Medical University. Seven fresh‐frozen human cadaveric cervical spine specimens from C4 to T1 were used in this study. The donors were all male with an average age of 44.3 years (range, 35–70 years). Lateral and anterior/posterior radiographs of the spine specimens were taken to exclude obvious neoplastic, traumatic, congenital conditions, and prior spine surgery. Bone mineral density (BMD) of the lumbar region of the specimens was measured using dual‐energy X‐ray absorptiometry. The average BMD was 1.031 g/cm,^2^ indicating none of specimens were osteoporotic. All specimens were placed in double‐layered plastic bags and stored at −20°C in a conservation cabinet until use. Each specimen was thawed to room temperature before testing. The musculature was dissected prior to use, but the ligaments, disks, and joint capsules were preserved. The upper third section of the C4 and T1 vertebrae were potted in dental stone mounts so that the C5/6 and C5/6 disks were horizontal.

#### Injured Model

Each injured specimen after right C6 hemi‐laminectomy and ipsilateral total C6 facetectomy served to simulate the bony defect after cervical dumbbell tumor resection through a unilateral posterior approach.

#### Eight Conditions of each Specimen

For pedicle screw placement, anatomical landmarks were used to locate the pedicle entry points and then a high‐speed drill was used to open an entry point to the cortex. The pedicle screws were then carefully inserted. The optimal screw length such that the screws were close to the anterior cortex of the vertebral body was determined in the preoperative planning of the screw trajectories. The screws were 26–32 mm long.^17^, ^18^

For lamina screw placement, the screw entry point was located at the beginning of the lamina from the spinous process at the midpoint of the dorsal arch. A high‐speed drill was used to enter the cortical window. Using a thin pedicle finder, the contralateral lamina was carefully drilled along its length with the pedicle finder aimed at the lateral mass. Procedures were performed under oblique cervical fluoroscopy (axial view of the lamina) to confirm that the pedicle finder did not violate the inner cortex of the lamina. Typically, the tip of the screw should not pass the medial margin of the lateral mass, and its orientation should be parallel to the slope of the lamina under anterior–posterior fluoroscopy. The length of screws was 18–22 mm.^19^

Transarticular screws were inserted using the Dal Canto technique. The ideal starting point was starting 2 mm caudal to the midpoint of the lateral mass and traveled 40° caudal and 20° lateral. Holes were prepared with a 2.5 mm drill and a 3.2 mm tap. The quadcortical transarticular screws were inserted through preserved channels into the C5/6 and C6/7 zygapophyseal joints. Screw lengths ranged from 20–22 mm.^10^

The iliac bone block for lateral mass reconstruction was harvested, shaped to the required size, and then inserted into the space between the C5 and C7 lateral masses on the affected side.

All instrumentations (Foshan Stable Surgical Implant, Guangdong, China) were made of titanium alloy (Ti‐6Al‐4 V). Hybrid fixations were inserted into each vertebra from C5 to C7 according to the experimental protocol of different instrumentation conditions, followed by the attachment of the rod for the pedicle screws and the lamina screws.

**Fig. 1:**
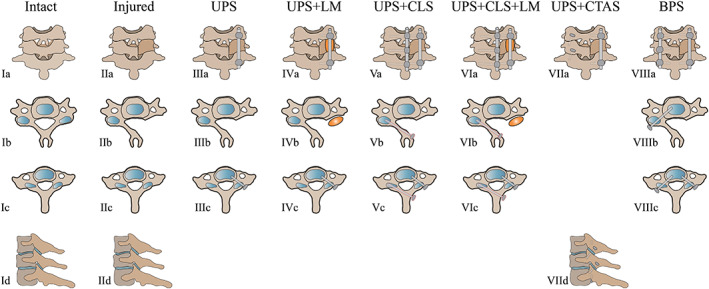
A schematic drawing of the eight different conditions of each specimen during testing. IA‐D Intact condition, posterior view, axis view, left lateral view; IIA‐D injured condition; IIIA‐C unilateral pedicle screw fixation (UPS); IVA‐C UPS fixation combined with lateral mass reconstruction (UPS + LM); VA‐C UPS fixation and contralateral lamina screw fixation (UPS + CLS); VIA‐C UPS + CLS fixation combined with lateral mass reconstruction (UPS + CLS + LM); VIIA, VIIID UPS fixation and contralateral transarticular screw fixation (UPS + CTAS); VIIIA‐VIIIC bilateral pedicle screw fixation status (BPS).

**Fig. 2:**
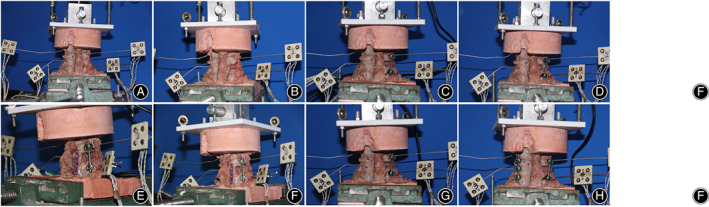
A posterior review of the eight different conditions of each cervical spine specimen during testing. (A) Intact condition, (B) injured condition, (C) unilateral pedicle screw fixation (UPS), (D) UPS fixation combined with lateral mass reconstruction (UPS + LM), (E) UPS fixation and contralateral lamina screw fixation (UPS + CLS), (F) UPS + CLS fixation combined with lateral mass reconstruction (UPS + CLS + LM), (G) UPS fixation and contralateral transarticular screw fixation (UPS + CTAS), (H) bilateral pedicle screw fixation status (BPS). The upper third section of the C4 and T1 vertebrae were potted in dental stone mounts.

The eight conditions of each specimen were shown in Figure 1 and Figure 2. And eight conditions of each specimen were tested, and the order 3–8 was randomized:

1. Intact condition.
2. Injured condition.
3. Unilateral pedicle screws (UPS) fixation.
4. UPS fixation combined with lateral mass reconstruction (UPS + LM).
5. UPS fixation and contralateral lamina screw fixation (UPS + CLS).
6. UPS + CLS fixation combined with lateral mass reconstruction (UPS + CLS + LM).
7. UPS fixation and contralateral transarticular screw fixation (UPS + CTAS).
8. Bilateral pedicle screw fixation (BPS).

#### Flexibility Testing

A custom‐designed and fabricated spine testing machine was used to apply a pure moment of ±2.0 Nm to the top vertebra, while allowing the specimen to move freely. The loading arm applied a continuous pure moment through two U‐joints and one linear bearing at a rate of 1°/s in all three primary loading directions: flexion‐extension, left/right lateral bending, and left/right axial rotation. The load was applied for three complete loading cycles. The first two cycles were used for preconditioning, and results of the third cycle were used for analysis. During flexibility test, the position of each vertebra was recorded by monitoring infrared light‐emitting diodes rigidly attached to each vertebra. A probe with four infrared light emitting diodes on the base of the spine machine defined a general anatomic specimen coordinate system. An optoelectronic camera system (Optotrak Certus; Northern Digital Inc., Waterloo, Ontario, Canada) was used to measure the three‐dimensional coordinates of the markers at a sampling frequency of 20 Hz. The range of motion (ROM) and neutral zone (NZ) between C5 and C7 in flexion‐extension, left/right lateral bending, and left/right axial rotation were calculated.

The motion tests were also randomized, and the nail trajectory was strengthened in order to minimize any deterioration of the specimens. All eight conditions of each specimen test were completed on the same day.

### Case Summary and Surgery

A 27‐year‐old female was admitted to the hospital after a C7/T1 spinal canal tumor was identified. She denied any prior medical problems, and stated she was in overall good health. She did not complain of any discomfort. Physical examination revealed normal cervical lordosis, no neck pain, no numbness of the limbs, normal muscle strength of the limbs, and a bilateral positive Babinski sign. Magnetic resonance imaging (MRI) showed a tumor the right intervertebral foramen of C7/T1, and its appearance suggested a benign lesion, most likely a neurogenic tumor (Figure 2: IA and IB).

The condition was discussed with the patient, and a detailed surgical plan was developed. After general anesthesia, the patient underwent posterior decompression of the right C7‐T1 spinal canal, tumor resection, internal fixation, and iliac bone graft placement.

For the operation, the patient was placed in the prone position. The right posterior superior iliac spine was exposed, and a 2.0 × 2.0 × 1.0 cm iliac bone block was obtained. The back of the neck was exposed, and a longitudinal incision in the middle of the back of neck was made and the tissue was dissected layer by layer to the right lamina of C6‐T1. An ultrasonic osteotome was used to remove the right hemivertebra and facet joints of C7‐T1. The ligamentum flavum was removed using a gun forceps, and some spinal dura mater and the tumor in the intervertebral foramen were exposed. After exposure, the tumor was found to be growing from the nerve root sleeve to the intervertebral foramen, with an intact capsule and clear margins with the surrounding tissues. The root of the tumor was tied off with No. 4 silk thread. Cauterization was performed with bipolar electrocoagulation, and the root sleeve was removed with a sharp knife and separation was performed at the junction between the tumor and the distal nerve root. The distal pedicle was cut off, and the tumor was completely removed. Appropriate lamina screws and pedicle screws were placed in C7 and T1, an iliac bone graft was placed between the lateral mass of C7‐T1 and the right pedicles, and titanium rods were placed to rebuild the stability of the lateral mass and facet joint column. The screw positions were checked by fluoroscopy, and then the screws were locked. After placing a drainage tube, the incision was in layers and the tumor tissue was sent for pathological examination.

### Statistical Analysis

Data are expressed as mean ± standard deviation. Differences in ROM and NZ among the different spine instrumentation conditions were analyzed using repeated measures analysis of variance at a 95% level of significance. Post hoc testing was performed using the least significant difference (LSD) test. Statistical analysis was performed with SPSS 25.0 software (SPSS Inc., Chicago, IL). Values of p < 0.05 were considered to indicate statistical significance.

## Results

### Biomechanical Study

**Fig. 3:**
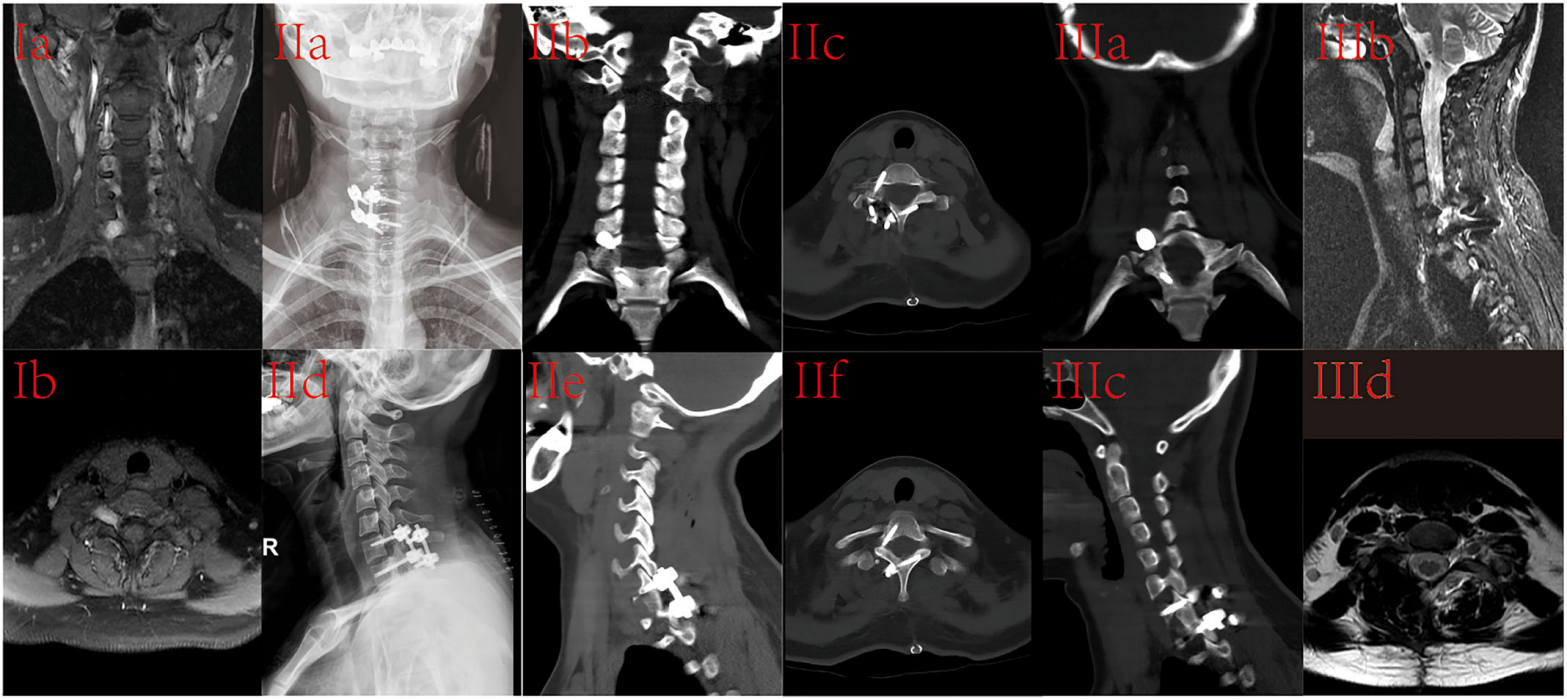
A 27‐year‐old female was diagnosed with a C7/T1 spinal tumor. The preoperative magnetic resonance imaging (MRI) coronal and cross‐sectional views (IA‐B) showed a tumor at the right intervertebral foramen of C7/T1. Anteroposterior and lateral radiographs (IIA, IID) and computed tomography (CT) coronal and sagittal views (IIB, IIE) 3 days after surgery showed the implant in place, and no fusion of the C7‐T1 lateral mass graft bone. Good positioning of the pedicle screws + pedicle screws in the C7 and T1 segments can be seen in transverse CT views (IIC, IIF). At 3 months after the operation, CT coronal and sagittal views (IIIA, IIIC) showed fusion of C7‐T1 lateral mass graft bone, and MRI coronal and cross‐sectional views (IIIB, IIID) showed complete resection of the tumor and no evidence of recurrence.

**Fig. 4:**
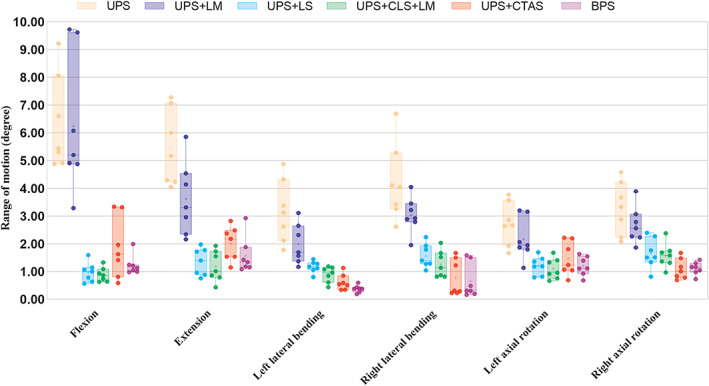
A grouped boxplot showing range of motion (ROM) of C5–C7 segment in flexion, extension, left lateral bending, right lateral bending, left axial rotation, and right axial rotation. The error bars represent one standard deviation. UPS: unilateral pedicle screw fixation; UPS + LM: UPS fixation combined with lateral mass reconstruction; UPS + CLS: UPS fixation and contralateral lamina screw fixation; UPS + CLS + LM: UPS + CLS fixation combined with lateral mass reconstruction; PS + CTAS: UPS fixation and contralateral transarticular screw fixation; BPS: bilateral pedicle screw fixation.

No screw loosening or fixation failure of any specimen was observed during the testing. The ROM and NZ between the C5 to C7 segments are shown in Figure 3 and Figure 4, respectively, and the measurement data are shown in Table 1 and Table 2, respectively.

**TABLE 1:** ROMs about different conditions between the C5‐C7(ROM，°，x ± s)

	Intact	Injured	UPS	UPS + LM	UPS + CLS	UPS + CLS + LM	UPS + CTAS	BPS
Flexion	13.64 (3.24)^a^, ^b^, ^c^, ^d^, ^e^, ^f^	14.35 (2.31)^a^, ^b^, ^c^, ^d^, ^e^, ^f^	6.35 (1.70)^a^, ^b^, ^c^, ^d^, ^e^, ^f^	6.24 (2.49)^a^, ^c^, ^d^, ^e^, ^f^	0.97 (0.35)^a^, ^b^	0.89 (0.25)^a^, ^b^	1.86 (1.10)^a^, ^b^	1.23 (0.35)^a^, ^b^
Extension	10.93 (3.05)^a^, ^b^, ^c^, ^d^, ^e^, ^f^	10.85 (3.37)^a^, ^b^, ^c^, ^d^, ^e^, ^f^	5.44 (1.36)^a^, ^b^, ^c^, ^d^, ^e^, ^f^	3.62 (1.32)^a^, ^c^, ^d^, ^e^, ^f^	1.35 (0.49)^a^, ^b^	1.19 (0.55)^a^, ^b^	2.01 (0.61)^a^, ^b^	1.57 (0.66)^a^, ^b^
Left lateral bending	8.77 (1.91)^a^, ^b^, ^c^, ^d^, ^e^, ^f^	8.99 (1.84)^a^, ^b^, ^c^, ^d^, ^e^, ^f^	3.17 (1.13)^a^, ^b^, ^c^, ^d^, ^e^, ^f^	1.98 (0.72)^a^, ^c^, ^d^, ^e^, ^f^	1.17 (0.21)^a^, ^b^, ^d^, ^e^, ^f^	0.89 (0.28)^a^, ^b^, ^c^, ^f^	0.61 (0.29)^a^, ^b^, ^c^	0.38 (0.13)^a^, ^b^, ^c^, ^d^
Right lateral bending	8.86 (2.11)^a^, ^b^, ^c^, ^d^, ^e^, ^f^	9.90 (2.38)^a^, ^b^, ^c^, ^d^, ^e^, ^f^	4.20 (1.38)^a^, ^b^, ^c^, ^d^, ^e^, ^f^	3.04 (0.64)^a^, ^c^, ^d^, ^e^, ^f^	1.57 (0.43)^a^, ^b^, ^d^, ^e^, ^f^	1.26 (0.48)^a^, ^b^, ^c^, ^f^	0.78 (0.66)^a^, ^b^, ^c^	0.0.65 (0.62)^a^, ^b^, ^c^, ^d^
Left axial rotation	7.38 (3.44)^a^, ^b^, ^c^, ^d^, ^e^, ^f^	9.96 (3.20)^a^, ^b^, ^c^, ^d^, ^e^, ^f^	2.73 (0.77)^a^, ^b^, ^c^, ^d^, ^e^, ^f^	2.17 (0.75)^a^, ^c^, ^d^, ^e^, ^f^	1.21 (0.33)^a^, ^b^	1.10 (0.38)^a^, ^b^, ^e^	1.47 (0.61)^a^, ^b^, ^d^	1.20 (0.34)^a^, ^b^
Right axial rotation	6.89 (2.14)^a^, ^b^, ^c^, ^d^, ^e^, ^f^	8.96 (2.13)^a^, ^b^, ^c^, ^d^, ^e^, ^f^	3.28 (0.95)^a^, ^b^, ^c^, ^d^, ^e^, ^f^	2.67 (0.67)^a^, ^c^, ^d^, ^e^, ^f^	1.66 (0.55)^a^, ^b^, ^e^, ^f^	1.58 (0.44)^a^, ^b^, ^e^, ^f^	1.09 (0.37)^a^, ^b^, ^c^, ^d^	1.11 (0.22)^a^, ^b^, ^c^, ^d^

*Note*: Data are presented as Mean (SD).

^a^ Statistical difference from Intact, or Injured, or UPS conditions (p < 0.05).

^b^ Statistical difference from UPS + LM condition (p < 0.05).

^c^ Statistical difference from UPS + CLS condition (p < 0.05).

^d^ Statistical difference from UPS + CLS + LM condition (p < 0.05).

^e^ Statistical difference from UPS + CTAS condition (p < 0.05).

^f^ Statistical difference from BPS condition (p < 0.05).

Abbreviation: ROM, range of motion; UPS: unilateral pedicle screw fixation; UPS + LM: UPS fixation combined with lateral mass reconstruction; UPS + CLS: UPS fixation and contralateral lamina screw fixation; UPS + CLS + LM: UPS + CLS fixation combined with lateral mass reconstruction; PS + CTAS: UPS fixation and contralateral transarticular screw fixation; BPS: bilateral pedicle screw fixation.

**TABLE 2:** NZs about different conditions between the C5‐C7(NZ，°，x ± s)

	Intact	Injured	UPS	UPS + LM	UPS + CLS	UPS + CLS + LM	UPS + CTAS	BPS
Flexion‐ extension	4.79 (3.36)^b^, ^c^, ^d^, ^e^, ^f^	5.05 (3.69)^b^, ^c^, ^d^, ^e^, ^f^	2.23 (0.86)^b^, ^c^, ^d^, ^e^, ^f^	1.18 (0.62)^a^, ^c^, ^d^, ^e^, ^f^	0.23 (0.13)^a^, ^b^, ^d^	0.16 (0.12)^a^, ^b^, ^c^	0.40 (0.34)^a^, ^b^	0.18 (0.05)^a^, ^b^
Lateral bending	3.49 (1.64)^a^, ^b^, ^c^, ^d^, ^e^, ^f^	3.89 (1.56)^a^, ^b^, ^c^, ^d^, ^e^, ^f^	1.31 (0.77)^a^, ^c^, ^d^, ^e^, ^f^	0.61 (0.44)^a^, ^d^, ^e^, ^f^	0.28 (0.08)^a^, ^c^, ^d^, ^f^	0.14 (0.06)^a^, ^b^, ^c^, ^f^	0.17 (0.17)^a^, ^b^	0.06 (0.04)^a^, ^b^, ^c^, ^d^
Axial rotation	2.01 (0.87)^a^, ^b^, ^c^, ^d^, ^e^, ^f^	3.06 (2.01)^a^, ^b^, ^c^, ^d^, ^e^, ^f^	0.52 (0.31)^a^, ^c^, ^d^, ^e^, ^f^	0.38 (0.18)^a^, ^b^, ^c^, ^d^, ^e^, ^f^	0.20 (0.06)^a^, ^b^	0.17 (0.04)^a^, ^b^	0.21 (0.13)^a^, ^b^	0.18 (0.05)^a^, ^b^

*Note*: Data are presented as Mean (SD).

^a^ Statistical difference from Intact, or Injured, or UPS conditions (p < 0.05).

^b^ Statistical difference from UPS + LM condition (p < 0.05).

^c^ Statistical difference from UPS + CLS condition (p < 0.05).

^d^ Statistical difference from UPS + CLS + LM condition (p < 0.05).

^e^ Statistical difference from UPS + CTAS condition (p < 0.05).

^f^ Statistical difference from BPS condition (p < 0.05).

Abbreviation: NZ, neutral zone; UPS: unilateral pedicle screw fixation; UPS + LM: UPS fixation combined with lateral mass reconstruction; UPS + CLS: UPS fixation and contralateral lamina screw fixation; UPS + CLS + LM: UPS + CLS fixation combined with lateral mass reconstruction; PS + CTAS: UPS fixation and contralateral transarticular screw fixation; BPS: bilateral pedicle screw fixation.

#### Comparisons of ROM

The ROM measurements of the C5‐C7 segments under eight conditions are shown in Table 1. Compared to the intact condition, the left axial rotation and right axial rotation ROM of the injured conditions were significantly increased (both, p < 0.05; Table 1). There were no differences in flexion, extension, left lateral bending, and right lateral bending ROM between these two conditions. The C5‐C7 ROM in all directions of the instrumented conditions UPS, UPS + LM, UPS + CLS, UPS + CLS + LM, UPS + CTAS, and BPS were significantly decreased compared to the intact condition and injured condition (all, p < 0.05).

Left lateral bending, right lateral bending, and right axial rotation ROM of the UPS + CLS + LM condition were significantly larger than that of the BPS condition (all, p < 0.05). However, flexion, extension, and left axial rotation ROM were similar between these two instrumentation conditions (all, p > 0.05). Except for left axial rotation and right axial rotation ROM (both, p < 0.05), there were no significant difference in flexion, extension, left lateral bending, and right lateral bending ROM between the UPS + CLS + LM condition and UPS + CTAS condition (all, p > 0.05). Flexion, extension, left axial rotation, and right axial rotation ROM were similar between the UPS + CLS + LM condition and the UPS + CLS condition (all, p > 0.05). However, left lateral bending and right lateral bending ROM of the UPS + CLS + LM condition were significantly reduced compared to the UPS + CLS condition (both, p < 0.05). The UPS + CLS + LM condition significantly reduced ROM in all directions compared to UPS condition and UPS + LM condition (all, p < 0.05).

There were no significant differences in ROM in all directions between the BPS condition and the UPS + CTAS condition (all, p > 0.05). Except for flexion direction (p > 0.05), the ROM in all other directions of the UPS + LM condition were significantly less than that of the UPS condition (all, p < 0.05).

#### Comparisons of the NZ

The NZ measurements of the C5‐C7 segments for eight conditions are shown in Table 2. There were no significant differences in the NZ in all directions between the intact conditions and the injured conditions (all, p > 0.05). Compared to the intact condition and injured condition, the C5‐C7 NZs of the instrumented conditions UPS, UPS + LM, UPS + CLS, UPS + CLS + LM, UPS + CTAS, and BPS were significantly decreased in all directions (all, p < 0.05).

The lateral bending NZ was significantly increased in the UPS + CLS + LM condition than in the BPS condition (p < 0.05), but there was no difference in the flexion‐extension and axial rotation NZ between the UPS + CLS + LM condition and BPS condition (both, p > 0.05). The NZ of the UPS + CLS + LM condition was the same as that of the UPS + CTAS condition in all directions (all, p > 0.05). In comparison to the UPS condition and UPS + LM condition, the NZs of the UPS + CLS + LM condition were significantly reduced in all directions (all, p < 0.05).

**Fig. 5:**
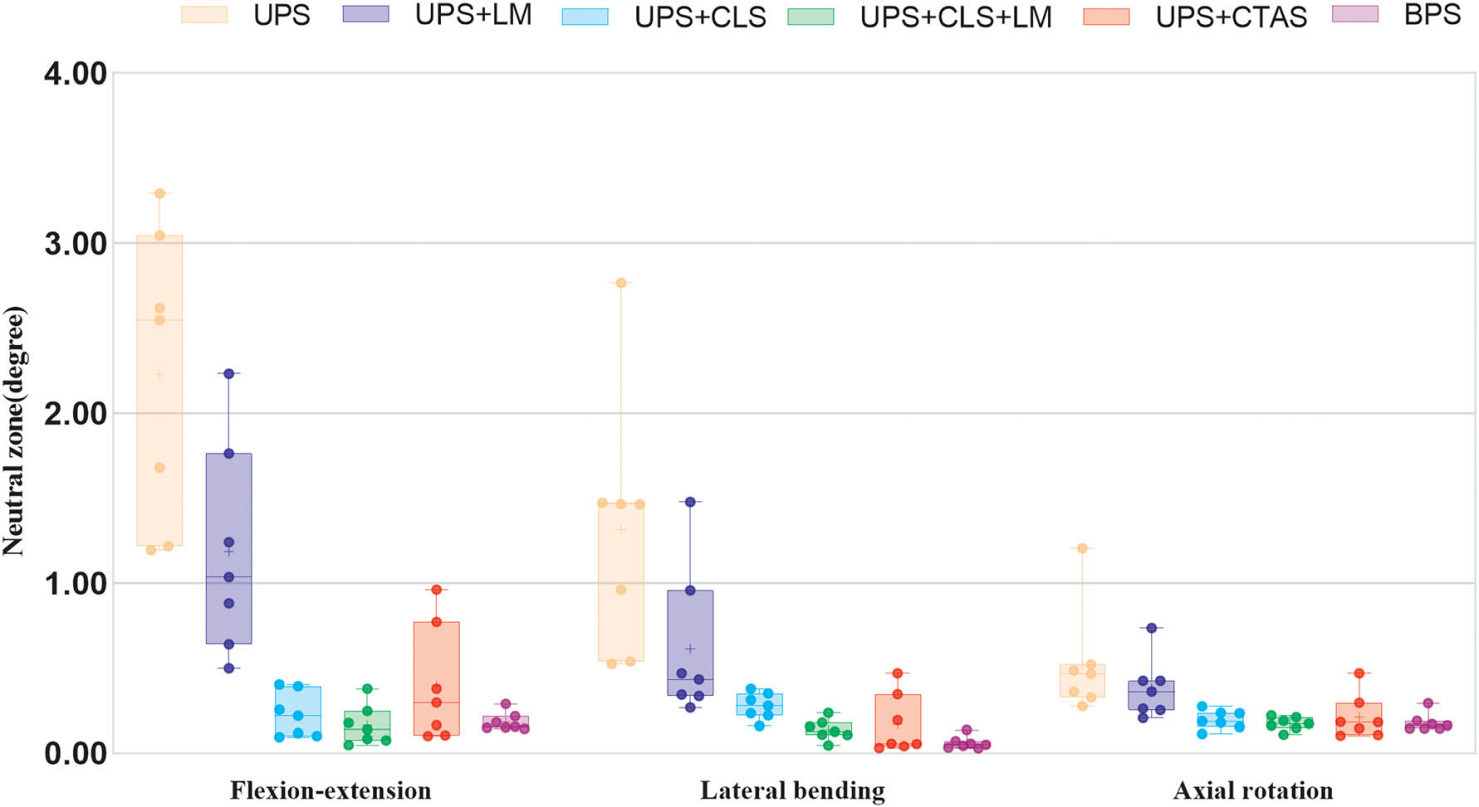
A grouped boxplot showing neutral zone (NZ) of C5–C7 segment in flexion–extension, lateral bending, and axial rotation. The error bars represent one standard deviation. UPS: unilateral pedicle screw fixation; UPS + LM: UPS fixation combined with lateral mass reconstruction; UPS + CLS: UPS fixation and contralateral lamina screw fixation; UPS + CLS + LM: UPS + CLS fixation combined with lateral mass reconstruction; PS + CTAS: UPS fixation and contralateral transarticular screw fixation; BPS: bilateral pedicle screw fixation.

The NZ in the axial rotation direction of the UPS + CLS + LM condition was significantly smaller than that of the UPS + CLS condition (p < 0.05), but similar to that of the UPS + CLS condition in the flexion‐extension and lateral bending direction (both, p > 0.05). There were no significant differences in NZ in all directions between the BPS condition and the UPS + CTAS condition (all, p > 0.05). The NZ in all directions of the UPS + LM condition were similar to those of the UPS condition (all, p > 0.05) (Figure 5).

### Case Outcome and Follow‐Up

Pathological examination of the tumor specimen was consistent with a Schwannoma. Anteroposterior and lateral radiographs (Figure 2: IIA, IID) and computed tomography (CT) coronal and sagittal views (Figure 2: IIB, IIE) 3 days after surgery showed the implant in place, and no fusion of the C7‐T1 lateral mass graft bone. Good positioning of the pedicle screws + pedicle screws in the C7 and T1 segments can be seen in transverse CT views (Figure 2: IIC, IIF).

At 3 months after the operation, the patient's sleep, diet, and bowel movements were normal and she was able to engage in moderate physical activity. CT coronal and sagittal views (Figure 2: IIIA, IIIC) showed fusion of C7‐T1 lateral mass graft bone, and MRI coronal and cross‐sectional views (Figure 2: III b, III d) showed complete resection of the tumor and no evidence of recurrence.

## Discussion

The main findings of the present study suggest that UPS + CLS + LM technique is a considerable technique compared to the traditional fixations. As for the flexion, extension, and left axial rotation stability, the UPS + CLS + LM condition was shown to be equivalent to the BPS condition in our biomechanical study. In terms of the left/right lateral bending stability, the UPS + CLS + LM condition was equivalent to the UPS + CTAS condition. In the right axial rotation direction, the UPS + CLS + LM condition was more stable than the UPS condition and UPS + LM condition. What's more, the internal fixation did not move, and the graft bone was seen with fusion in the patient treated with this technique following resection of a dumbbell tumor via a unilateral posterior approach.

### Biomechanical Stability of Unilateral Pedicle and Lamina Screw Fixation with Lateral Mass Reconstruction

The biomechanical results of this study demonstrate that single‐level hemi‐laminectomy and facetectomy of intact cervical spine can impair the stability of left/right axial rotation. Because the ROM of the injured condition was larger than that of the intact condition after C6 hemi‐laminectomy and facetectomy. Therefore, the internal fixation is necessary after resection of a unilateral dumbbell tumor via a posterior approach.

The ROM comparisons showed that the stability of the UPS + CLS + LM condition was sufficient in all directions. Firstly, the biomechanical study results showed that the flexion, extension, and left axial rotation stability of the UPS + CLS + LM condition were the same as those of the UPS + CTAS condition and BPS condition. This indicated that the UPS + CLS + LM condition performed well in the flexion‐extension direction. Secondly, the lateral bending stability of the UPS + CLS + LM condition was the same as that of the UPS + CTAS condition. These two techniques had good stability against lateral bending because the lateral mass and facet column, where the graft bone and transarticular screws were placed, were away from the center of the vertebral body. Not surprisingly, the UPS + CLS + LM condition was weaker in lateral bending stability than the BPS condition because the BPS technology was a three‐column fixation. Thirdly, the right axial rotation stability of the UPS + CLS + LM condition was less than that of the UPS + CTAS condition and BPS condition. Due to the additional fixation of the contralateral lamina and the reconstruction of the lateral mass on the affected side, the UPS + CLS + LM condition had a better ability to resist axial rotation on the right side (the side where hemi‐laminectomy and facetectomy were performed) compared to the UPS condition, the UPS + LM condition, and the UPS + CLS condition. However, the contralateral pedicle screw of the BPS condition fixed the three columns to have a longer force arm. The contralateral transarticular screw of the UPS + CTAS condition also fixed the facet column on healthy side by penetrating four layers of cortical bone. Therefore, the UPS + CLS + LM condition was not the most stable of the eight conditions in the right axial rotation.

Lateral mass reconstruction was found to improve lateral bending stability by comparing the stability of the UPS + CLS + LM condition with the UPS + CLS condition in our single‐level hemi‐laminectomy and facetectomy model. Because lateral mass reconstruction supported the middle column damaged by the facetectomy, the lateral bending ROM of the affected segment was limited. However, Ji et al. compared the stability of the unilateral pedicle and lamina screws fixation combined with graft bone implant condition and the unilateral pedicle and lamina screws fixation condition in a two‐level hemi‐laminectomy and facetectomy model in a biomechanical test, and found that the addition of a lateral mass improved anti‐rotation stability.^20^ The reason for this may be that the longer the injured segment, the poorer the axial rotational resistance. Lateral mass reconstruction across a two‐level spine provides both a direct connection to the middle column and increased interfacial friction with the adjacent segmental spine, thus improving the rotational resistance. According to the above views, lateral mass reconstruction repairing the middle column of spine improves cervical stability in different directions for different ranges of hemi‐laminectomy and facetectomy.

### The Clinical Significance of Unilateral Pedicle and Lamina Screw Fixation with Lateral Mass Reconstruction

The use of lateral mass reconstruction in addition to fixation with a rod‐screw system is worth considering when the middle column and posterior column of the injured segment are incomplete. Since the risk of screw loosening, fracture, or displacement is increased by the load transmitted over the adjacent segment through the rod‐screw system, lateral mass reconstruction reduces the load on the rod‐screw system and therefore reduces the occurrence of cervical instability when used to repair a cervical vertebral injury.

Posterior internal fixation improves the fusion rate and eliminates the need for strict external fixation and as such pedicle screws and lateral mass screws are widely used to treat spinal instability as an alternative to posterior external fixation^21^, ^22^ There are two main types of internal fixation commonly used in the cervical spine, unilateral fixation and bilateral fixation techniques. With respect to the amount of surgical trauma and complications, unilateral fixation preserves the contralateral bone structure and soft tissue and thus there is less surgical trauma, but the risk of instability is higher^3^, ^23^, ^24^, ^25^ The biomechanical stability of the cervical spine is better with bilateral fixation; however, more soft tissue and bone structures need to be dissected or removed, which increases the surgical trauma, and there is a greater risk of neurovascular damage.^26^, ^27^, ^28^ Shi et al. investigated the stability of unilateral pedicle screw and lamina screw fixation using a lower cervical instability model, and reported that the stability of unilateral pedicle screw and lamina screw fixation was similar to that of bilateral pedicle screw fixation in flexion, extension, and rotation, but was different in lateral bending.^29^ Previously, Ji et al. conducted a comparative biomechanical study of unilateral pedicle screw and lamina screw fixation with lateral mass reconstruction for treatment of two‐level hemi‐laminectomy and facetectomy of the cervical spine. The result showed that the stability of this technique was better than that of unilateral pedicle screws, and closer to that of bilateral pedicle screws.^20^ In our biomechanical study, the stability of different instrumentation techniques was compared after single‐level hemi‐laminectomy and facetectomy of the cervical spine. Except right axial rotation, the stability of UPS + CLS + LM condition in other directions was similar to that of BPS or UPS + CTAS condition. And this condition in all directions was more stable than the UPS condition and UPS + LM condition. Therefore, unilateral pedicle screw and pedicle screw fixation + lateral mass reconstruction combines the advantages of unilateral fixation and bilateral fixation to improve stability in hemi‐laminectomy and facetectomy cervical spine.

Unilateral pedicle screw and lamina screw fixation with lateral mass reconstruction was used to reconstruct C7‐T1 stability in our patient, and satisfactory bone fusion was achieved at 3 months after surgery without any significant complications.

### Strengths and Limitations

The hemi‐laminectomy and facetectomy destroy the posterior structure of the cervical spine, which may lead to the progression of cervical instability and cervical kyphosis, thus internal fixation and fusion are sometimes required. We not only carried out a biomechanical experiment but also reported a case about the short‐term clinical effects of this technique, so that the conclusions of this fixation technique drawn from the study can provide convincing references for clinical treatment. What's more, six instrument conditions have been tested on the same specimens in the biomechanical experiment to provide an exhaustive comparison and provide an important reference value for relevant fixation techniques.

There are limitations to this study that need to be considered. The same specimen needs to be repeatedly loaded and unloaded, and the fixation method needs to be changed several times during the biomechanical testing. However, in this study the order of testing the different fixation methods was randomized, and the nail path was strengthened when changing fixation methods to reduce the influence on the test results. In this study we used isolated spine specimens to simulate ideal bone and ligament structure and ignored the role of muscle tissue for biomechanical stability testing; thus, the results only reflect the effect of fixation in the short term after surgery. Further study to examine the long‐term effects, such as fatigue testing, are needed. A minimally invasive approach combining strong fixation with a unilateral pedicle rod system and elastic fixation with contralateral pedicle screws should theoretically result in good bone fusion, reduced contralateral tissue damage, and delayed degeneration of the adjacent segment. However, we only reported the results of one actual case, and no comparative study with BPS was performed. Thus, the value of this fixation technique with respect to promoting bone fusion and delaying degeneration of adjacent segments needs further evaluation.

## Conclusion

After resection of a unilateral dumbbell tumor in the cervical spine, unilateral pedicle screw and lamina screws fixation with lateral mass reconstruction is a reliable method that provides sufficient immediate stability and promotes bone fusion. The method also reduces surgical trauma and complications.

## Author Contributions

Yongqiang Zeng: Methodology, Investigation, Formal Analysis, Writing—original draft. Zhiping Huang: Methodology, Validation, Investigation. Zucheng Huang and Yongquan Cheng: Validation, Investigation; Qing'an Zhu: Conceptualization, Methodology; Hui Jiang and Wei Ji: Conceptualization, Writing—Review & Editing, Supervision, Project Administration. All authors listed meet the authorship criteria according to the latest guidelines of the International Committee of Medical Journal Editors. And all authors are in agreement with the manuscript.

## Conflicts of interest

The authors declare that they have no personal, financial, or institutional interest and ethical/legal conflicts involved in this article.

## Funding information

NA.
